# Standing and Special Committees

**Published:** 1867-07

**Authors:** 


Dr. C. Goodbrake, in behalf of the Committee on Nominations, reported the following as standing and special committees to report at the next annual meeting of the Society:—

*Committee on Practical Medicine and Epidemics*—Drs. H. A. Johnson, of Chicago; E. P. Cook, of Mendota; and Wm. Massey, of Grandview.

*Committee on Surgery*—Drs. E. Powell, of Chicago; Geo. T. Allen of Springfield; and S. T. Trowbridge, of Decatur.

*Committee on Obstetrics*—Drs. E. W. Moore, of Decatur; G. W. Albin, of Neoga; and W. A. Elder, of Bloomington.

*Committee on Drugs and Medicines*—Drs. Henry Wing, of Collinsville; W. S. Edgar, of Jacksonville; and D. S. Jenks, of Plano.

*Committee on Ophthalmology*—Drs. H. H. Roman, of Springfield; Joseph S. Hildreth, and E. L. Holmes, of Chicago.

*Committee on the Causes, Pathology, and Treatment of Cholera*—Dr. N. S. Davis, of Chicago.

*Committee on the Language of the Pulse*—Dr. J. H. Hollister, of Chicago.

*Committee on Fracture of Lower End of Radius*—Dr. David Prince, of Jacksonville.

*Committee on Conservatism in the use of Remedial Agents*—Dr. E. Ingals, of Chicago.

On motion of Dr. H. A. Johnson, Quincy was designated as the place for holding the next annual meeting of the State Medical Society.

On motion, the report of the Nominating Committee was adopted.

The resolution offered by Dr. J. A. Allen, in relation to the use of fruit during seasons when cholera is prevalent, being the special order, was taken up; Dr. Prince having the floor.

Dr. Prince advocated the resolution, and gave some interesting facts relating to the efforts to suppress the use of fruits in St. Louis during a former prevalence of cholera in that city.

Dr. W. S. Edgar thought some fruits might be used safely, while others were dangerous. Hence, he moved to amend the resolution by inserting the words "some fruits," which was lost.

Dr. E. Ingals offered the following as a substitute for the resolution of Dr. Allen, but subsequently modified his motion, so as to make it an additional resolution:—

"*Resolved*, That the diet that is ordinarily most wholesome is the proper diet to be taken during an epidemic of cholera."

After further discussion by Drs. T. F. Worrell, D. W. Young, and H. A. Johnson, Dr. N. S. Davis moved to amend the original resolution, by inserting after the word "fruits," the words "taken at the ordinary meals." He fully endorsed the position that good, ripe fruits not only did not predispose to attacks of cholera, but were positively beneficial as articles of food. But, like all other articles of food, they should be taken only at proper intervals; and, as physicians, they should not adopt any resolution that might be so construed as to encourage the eating of even ripe fruit at any and all hours of the day. The amendment was accepted without opposition.

Dr. H. A. Johnson thought it injudicious to adopt any resolution on the subject, and moved to lay the resolutions on the table. The motion was lost, by 29 yeas, 34 nays.

The resolution offered by Dr. Allen, and amended, was then adopted as follows:—

*Resolved*, That the moderate use of ripe, but not stale or decayed, fruits, taken at the ordinary meals, is not objectionable, as tending to produce cholera, but, rather, conducive to the preservation of health during the hot season.

The resolution offered by Dr. E. Ingals, was also adopted.

Dr. J. H. Hollister offered the following resolution, which was adopted:—

*Resoleed*, That a special committee of three be appointed upon the subject of Necrology, having reference to the proper preservation of statistical and other memorial records of its deceased members.

Dr. Hollister also proposed such an alteration of the Constitution as would make the Committee on Necrology a *standing* committee. Laid on the table until the next annual meeting.

Dr. J. S. Whitmire offered the following, which was adopted:

*Resolved*, That it now be made a permanent rule of this Society, that no member shall be permitted to speak a second time on any one subject, without the special permission of this Society.

Dr. E. W. Moore moved that Art. 4 of the Constitution be so amended that the term of office of the President and Vice-Presidents shall commence at the opening of the next annual meeting after their election. Laid over, under the rules.

The hearing of reports of standing committees was then resumed, and Dr. L. T. Hewins, one of the Committee on Practical Medicine, read an interesting report, which was accepted and referred to the Committee on Publication.

Dr. DeLaskie Miller, Chairman of the Committee on Obstetrics, presented an abstract of his report, which, on motion, was accepted, and the report referred to the Committee on Publication.

Dr. H. W. Davis presented a lengthy report, embodying the results of his surgical practice during his recent service in the army; which was accepted without reading and referred to the Committee on Publication.

Dr. Geo. T. Allen presented a short paper on the Radical Cure of Inguinal Hernia, with the instruments required for the operation. The paper was referred to the Committee on Publication.

Dr. Trowbridge, Chairman of the Committee appointed at the last annual meeting, to urge the enactment of suitable laws by the State Legislature, reported as follows:—

